# Cost of physiotherapy non-attendance at a metropolitan hospital in Australia: A time-driven activity-based costing study

**DOI:** 10.1136/bmjopen-2023-083420

**Published:** 2025-05-24

**Authors:** Shayma Mohammed Selim, Sundresan Naicker, Sanjeewa Kularatna, Hannah E Carter, Samantha Borg, Codie Armstrong, Melanie Walkenhorst, Brittney Kunst, Steven M McPhail

**Affiliations:** 1 Australian Centre for Health Services Innovation and Centre for Healthcare Transformation, School of Public Health and Social Work, Queensland University of Technology, Kelvin Grove, Queensland, Australia; 2 Health Services and Systems Research, Duke-NUS Medical School, Singapore; 3 Logan Physiotherapy Department, Metro South Hospital and Health Service, Logan, Queensland, Australia; 4 Digital Health and Informatics Directorate, Metro South Hospital and Health Service, Woolloongabba, Queensland, Australia

**Keywords:** Hospitals, Health economics, Health Services

## Abstract

**Abstract:**

**Objectives:**

(1) Identify the processes, staff time and labour costs associated with non-attendance at two physiotherapy outpatient clinics using time-driven activity-based costing; (2) estimate labour cost-burden of non-attendance response scenarios.

**Design:**

A six-step time-driven activity-based costing method was used, including scenario analyses.

**Setting:**

Two tertiary hospital outpatient clinics.

**Participants:**

Clinic non-attendance rates were determined from digital administrative records for participating clinics. Interviews and iterative discussions were conducted with 15 administrative and clinical staff to establish process maps and key parameters.

**Primary and secondary outcome measures:**

The primary outcome was health service labour cost associated with clinic non-attendance. Four key work processes were identified and costed (2023, A$).

**Results:**

Clinic non-attendance rates for the 2018–2021 period were 8% (Clinic 1) and 10% (Clinic 2). Complex triaging cases constituted greater costs than simple triaging cases. Projected annual costs of non-attendance were as high as A$114 827 for a single clinic. The most expensive referral and response scenario was internal referral with non-attendance that was converted to a telephone appointment (mean cost of A$113/appointment).

**Conclusion:**

Non-attendance rates at participating clinics were at the lower end of values reported in prior literature; however, substantial healthcare resource waste was still evident. Findings highlighted the extent to which non-attendance at scheduled clinic appointments may not only impact patients’ welfare through lost treatment opportunity, but also carry substantial opportunity cost from wasted hospital resources that could have been allocated to other referred patients. Establishing the effectiveness and cost-effectiveness of interventions to reduce non-attendance remains a priority.

**STRENGTHS AND LIMITATIONS OF THE STUDY**

- This study used time-driven activity-based costing, which can be an effective and efficient approach for identifying healthcare labour costs and improving operational efficiency.
- This study was conducted at publicly funded physiotherapy clinics and as such, findings may not be fully generalisable to other contexts.
- The scope of this study was limited to understanding personnel labour costs (productivity costs) as the resource use associated with each activity of a process.

## Introduction

 Amidst rising costs of healthcare delivery, constrained healthcare resources and burgeoning demand for services, there is an increased need to understand areas for resource waste reduction.^1^ In contemporary healthcare systems, non-attendance, defined as instances when patients fail to attend scheduled healthcare appointments, is a significant waste of financial resources.^2^ Although there is no current robust national estimate of wasted healthcare resources attributable to non-attendance in Australia, there have been compelling estimates arising from healthcare systems internationally. For example, in the UK, the projected non-attendance costs across the entirety of the healthcare system are estimated to be £912 million per annum.^3^ Estimated non-attendance costs across the US healthcare system equate to US$150 billion per annum.^4^

Physiotherapy is an important allied health profession with a pivotal role in hospital settings that typically spans a broad range of conditions and treatments.^5^ In hospital outpatient contexts, this includes the provision of interventions that serve as conservative alternatives to more invasive and costly interventions, including surgeries, as well as presurgical and postsurgical interventions.^5 6^ Non-attendance at physiotherapy clinics directly contributes to lost clinical time, which can equate to wasted resources, if staff are unable to redirect the appointment time to other productive healthcare-related activities.^2 7 8^ Poor clinic attendance or multiple failed attendances are likely to directly impact patient outcomes.^9 10^ Non-attendance also results in vacant appointment slots, which can contribute to ongoing problems or further deterioration of outcomes for patients who remain on waiting lists.^9^ This is concerning in light of lengthy wait lists, particularly where target wait list timeframes are being exceeded.^11^

A recent scoping review identified highly variable rates of non-attendance at health appointments in international published literature, ranging from 9% to 43%.^12^ Two studies identified in that review reported non-attendance rates in Australia. Both studies were specific to dental clinics, with one identifying a non-attendance rate of 9.3%,^13^ while the other identified non-attendance at 5% among adults and 11% among children.^14^ Yet, even with these non-attendance rates, the costs to the health system have received little exploration in the existing literature.^2^ Furthermore, it has been recognised that to foster improved value in healthcare, when conceptualised as health outcomes achieved per dollar spent, it is imperative to first understand where resource waste is occurring.^15^

There are multiple costing methods that can be used to estimate non-attendance. Time-driven activity-based costing (TDABC) has commonly been used by healthcare providers to estimate service delivery costs.^16^ Several studies have applied the TDABC method to healthcare pathways, including arthroplasty,^17^ radiology,^18^ paediatric specialties^19 20^ and rehabilitation.^21^ TDABC and its application in healthcare allow for an understanding of granular activities involved in a clinic’s key workflow processes, estimates of time associated with conducting activities constituting each process, and associated costs.^22 23^ This provides the opportunity to obtain insights that can inform ways to improve operational efficiency.^22 23^ The TDABC method primarily requires the identification of discrete activities and the estimation of two parameters (unit of resources and time required) to calculate resource cost for each activity. First, the cost of a unit of resources (eg, per-minute cost of personnel time) and second, the time required to perform each activity of a process (eg, 30 min).^16 22 23^

Physiotherapy clinics were chosen in this study as potential use case examples of a broader non-attendance issue within the health system at large, given their increasing role in mitigating invasive surgical procedures and managing a range of chronic health conditions.^5 6^ As such, this study seeks to address two specific aims. First, using TDABC to identify the processes, staff time, and labour costs from the health service perspective associated with responding to non-attendance at two physiotherapy outpatient clinics. Second, to estimate the specific labour cost burden of different referral sources and response scenarios relating to non-attendance for the clinics over a year.

## Methods

### Study design

**Figure 1:**
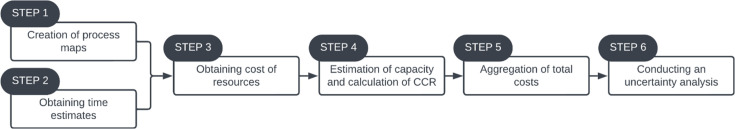
Time-driven activity-based costing steps. CCR, Capacity Cost Rate.

This study used adapted TDABC steps from Kaplan and Anderson^24^ (presented in figure 1) that included six discrete steps. The steps were: (1) process mapping discrete activities conducted by staff associated with rostering and managing incoming patients, (2) obtaining time estimates for each activity of the identified processes, (3) obtaining the labour cost for resources used, (4) estimation of the practical capacity of each resource and calculation of capacity cost rate, (5) aggregation of total labour costs through the synthesis of cost and time data and (6) conducting an uncertainty analysis simulation to assess the fidelity of cost estimates. The results from the TDABC were then used to project costs to address the second study aim. It is important to note that the study intentionally focused on labour costs, as the incremental costs of other resource types, for example, consumables, were considered by participating clinics to be negligible in the context of their high levels of digital maturity where overarching non-labour system costs were unlikely to be impacted by rates of attendance.

### Study setting and context

This study was conducted over 12 months, from July 2022 to July 2023, across two physiotherapy outpatient clinics co-located within a single hospital. The hospital is located within Metro South Health and Hospital Service in Queensland, Australia, which serves an estimated 1.2 million people in its designated catchment area.^25^ The first clinic (Clinic 1) specialises in musculoskeletal physiotherapy, is staffed with five physiotherapists and receives on average 2997 patient referrals annually. The second clinic (Clinic 2) specialises in women’s, men’s and pelvic health physiotherapy. This clinic is staffed by six physiotherapists and receives on average 3347 patient referrals per year. Both outpatient clinics are supported by the same 4 administrative staff.

### Study participants

In total, 15 staff across the two physiotherapy clinics participated across different study phases, as appropriate. They were recruited using a snowballing strategy. Clinic staff included the outpatient physiotherapy department director (n=1), clinic physiotherapists (n=11, including 2 who were team leaders) and administrative staff (n=4, including 1 who was the administration manager). All staff consented to be involved in the study. This study was approved by the Metro South Health Human Research Ethics Committee (HREC/2021/QMS/81605).

### Patient and public involvement

Patients were not involved in the design, conduct, reporting or dissemination plans of this research.

### Data collection and synthesis

The process of collecting data and analysing information was concurrent. An initial meeting was conducted at the beginning of the study with key stakeholders, which included the physiotherapy outpatient department director (n=1), team leader physiotherapists for both outpatient clinics (n=2) and the administration manager (n=1). This meeting facilitated the sharing of study data collection documents by the research team and allowed clinic staff to provide a brief overview of their processes relating to non-attendance. This information was used to inform a series of facilitated discussions described below. This discussion and all subsequent discussions were audio recorded and transcribed by a study researcher (SMS).

#### Steps 1 and 2: Process map creation and obtaining consensus time estimates

The first two steps (process map creation and obtaining time estimates) were conducted concurrently. This involved two semi-structured facilitated discussions conducted via videoconference with key stakeholders (n=3) to create process maps and obtain time estimates. This was followed by a validation activity conducted via email correspondence with the broader staff team from both clinics, which included 11 physiotherapists and 4 administrative staff. All participants were involved in at least one of the following clinic procedures: (1) operationalising the clinic referral process, (2) appointment scheduling and (3) provision of care to patients. The first facilitated discussion focused on understanding key workflow processes involved in service delivery for physiotherapy patients. Key workflow processes are a broad range of procedures conducted by the clinics, from which a set of discrete activities can be performed in combination or sequence to achieve a specific outcome.^23^ For example, all activities completed by administrative staff that form appointment booking for patients (eg, calling the patient, confirming a suitable appointment time). Following this discussion, a study researcher (SMS) inductively analysed audio transcripts and used information from Step 1 to create initial workflow process maps.

The second facilitated discussion focused on verifying the key workflow processes and corresponding activities outlined in the process map. A time estimate for each activity was simultaneously obtained during this verification process. Clinic staff were asked to estimate how long (in minutes) each activity took (eg, time taken by an administrative staff member to book an appointment) and reach a group consensus on each estimate. To account for variability in time taken for specific activities, participants also discussed and agreed on a minimum time and maximum time to complete each activity. Subsequently, process maps were finalised with time estimates and presented for validation to the broader clinic staff team. To conduct the validation activity, we used a think-aloud method to ensure that the data used in TDABC calculations were appropriate and represented up-to-date organisational practices.^26^ The time estimates elicited for each activity and associated minimum/maximum values were presented in a table format, to aid in their comprehensibility when think-aloud^26^ took place, while the process maps were represented as visual figures for a more accessible interpretation. All participants were asked to verify if any component was either missing or inaccurate. Following this collaborative review, a study researcher (SMS) made any necessary adjustments to both the process maps and their corresponding time estimates, verbally relaying this information back to participants for a final round of verification. The same study researcher (SMS) then finalised both process maps and the corresponding time estimates. The interview guide detailing the types of questions that were asked during the creation of the process maps and obtaining time estimates can be found in the online supplemental appendix.

#### Step 3: costing resources

Resources were costed based on Australian currency (A$) for July 2023 and were relevant to the health service where the study was being conducted. Personnel labour rates were calculated using the mid-point of the 2023 award rates for the state of Queensland for physiotherapists and administrative staff.^27^ The labour cost estimates included salary and an additional 25% on-costs to account for leave and other entitlements including superannuation funded by the health service.

#### Step 4: Estimation of the practical capacity and capacity cost rate

Staff theoretical capacity assumes that 100% of staff time is dedicated to the work role.^24^ Practical capacity accounts for breaks, training and other activities unrelated to service delivery.^24^ The current study calculated a practical capacity of 80% of the theoretical capacity to account for such activities being undertaken by staff as part of their roles. This estimate aligns with recommendations from Kaplan and Anderson.^24^ The practical capacity cost rate for personnel (A$/min) was calculated as the full expense for the year (A$/year) divided by the practical capacity (total number of minutes per year). These costs are described in online supplemental table 1.

#### Step 5: Aggregation of total costs

Costs were aggregated for each individual activity that constituted a broader process (eg, referral, booking, reminder and appointment attendance) where an associated resource was used, and time was required for the activity. To calculate the total cost, the capacity cost rate of each resource was multiplied by its duration of use in each activity of the process.

#### Step 6: Uncertainty analysis

Monte Carlo simulations were performed to account for uncertainty associated with the TDABC model inputs (time estimates and labour cost) used in estimating the total costs generated in Step 5. To reflect uncertainty surrounding the values used for estimates of staff time for conducting different activities and the varying labour costs for different personnel, a probability distribution was assigned to each parameter. In this study, a normal distribution was assumed for each parameter. ShinyPrior, a web-based application for estimating probability distributions from summary statistics, was used to convert estimates for each model input elicited during the facilitated discussion into a distribution (mean and SD) that could be used in the simulations.^28^ The Monte Carlo simulations were conducted using 1000 iterations, in which values were randomly sampled from the probability distribution constructed for each model input to generate simulated observations. These simulated observations were then used to estimate the mean and 95% CIs for the total cost of each activity.

### Scenario analysis and cost projections

The results from the TDABC were used to conduct an analysis constituting different referral sources and staff response scenarios related to non-attendance at the physiotherapy clinics and to project costs associated with these paths over a year. The scenario analysis combined several discrete activities related to 30 possible referral source and response scenarios. The cost projections used non-attendance rates established for each clinic from digital administrative data sources for the period of 2018–2021 as the basis for determining costs for each scenario. All analyses, including Monte Carlo simulations, were performed in Microsoft Excel.

## Results

### Workflow processes

**Figure 2:**
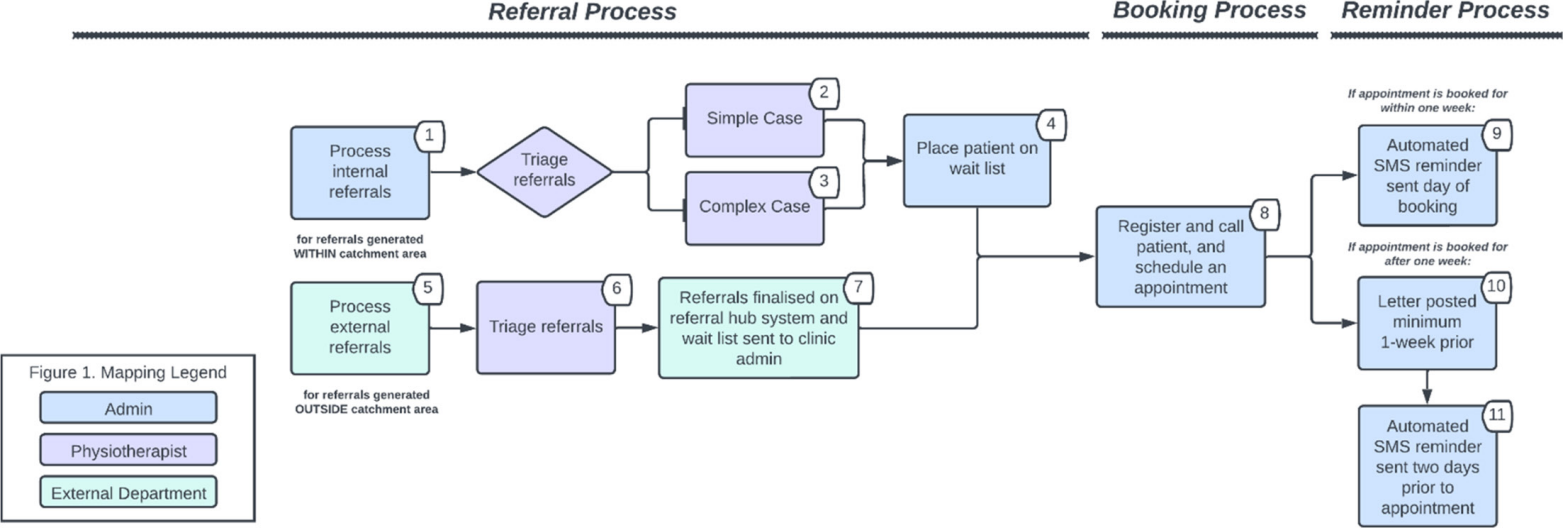
Workflow map of referral, booking and reminder process. Displayed numbers correspond to activity numbers in table 1. SMS, short message service.

The physiotherapy clinics had four key work processes where personnel labour is allocated: referrals, booking, reminders and appointment attendance (relating to when a patient attends, fails to attend, or cancels an appointment). These processes are outlined in figures2  3. The activity time and corresponding personnel responsible for conducting each activity are presented in table 1.

**Figure 3:**
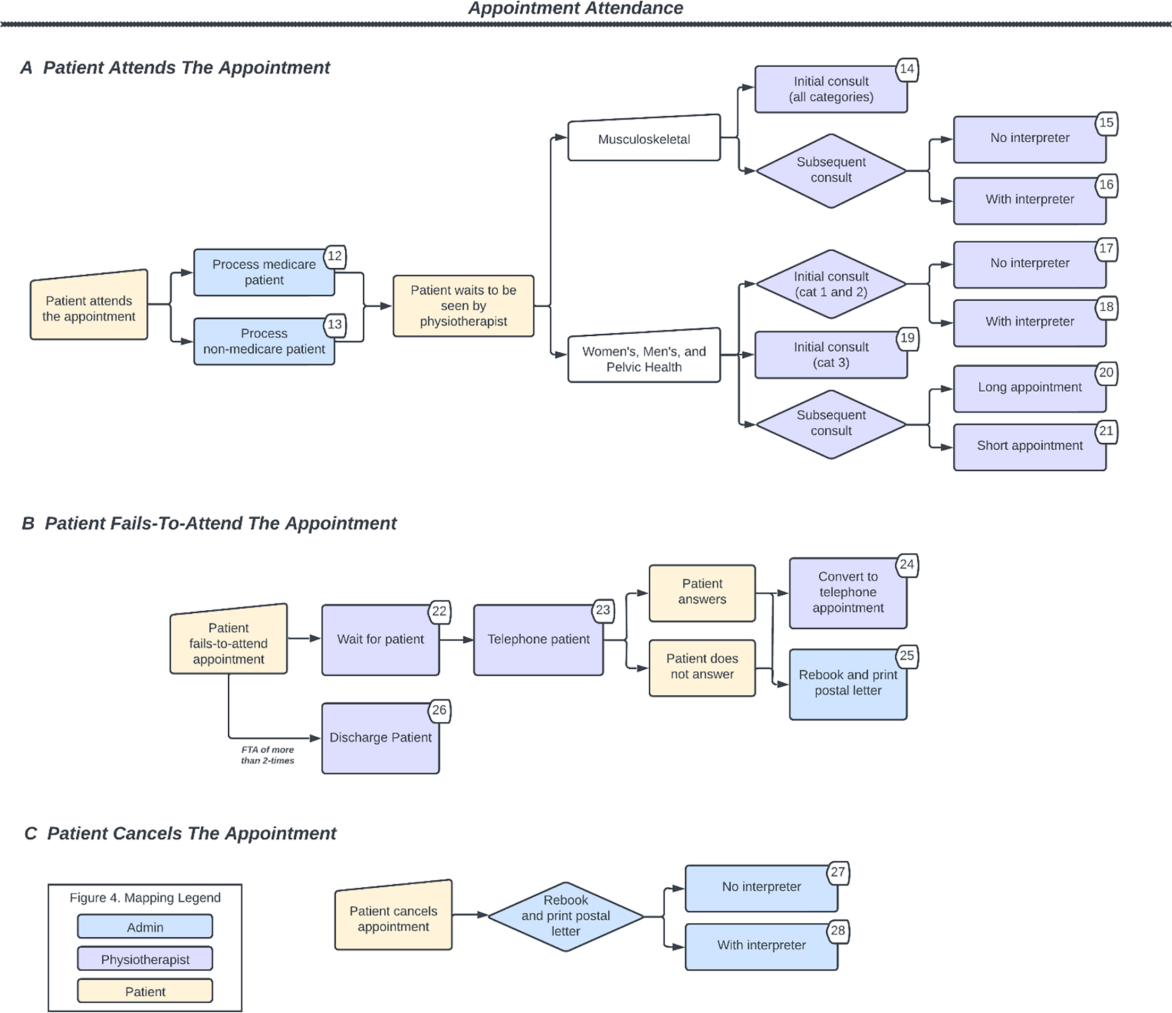
Workflow map for when a patient attends, fails-to-attend (FTA) and cancels a scheduled appointment. Displayed numbers correspond to activity numbers in table 1; categories (abbreviated as CAT) refer to triage categories.

**Table 1:** Activity and 2023 cost breakdown of physiotherapy clinic processes

#	Activity Description		Activity Time (minutes)	Capacity Cost Rate (A$ per minute)	Monte Carlo Simulations		
					Total Cost (A$)*		
			Mean (95% CI)	Mean (95% CI)	Mean	SD	95% CI
	**Referral process**						
	***Internal referrals***						
1	Process internal referrals that arrive via the internal hospital referral system, fax, or email		4.75 (3.80 to 5.70)	A: 1.01 (0.81 to 1.21)	4.80	0.67	3.54 to 6.17
2	Triage referrals into category 1, 2 and 3	Simple case	2.00 (1.00 to 3.00)	P: 1.48 (1.18 to 1.77)	2.95	0.83	1.40 to 4.66
3		Complex case	17.50 (5.00 to 30.00)	P: 1.48 (1.18 to 1.77)	26.04	10.00	7.77 to 46.98
4	Place patient on wait list		5.00 (4.00 to 6.00)	A: 1.01 (0.81 to 1.21)	5.03	0.70	3.78 to 6.52
	***External referrals***						
5	Process external referrals		4.75 (3.80 to 5.70)	RH: 1.01 (0.81 to 1.21)	4.83	0.68	3.53 to 6.20
6	Triage referrals into category 1, 2 and 3		6.00 (4.00 to 8.00)	P: 1.48 (1.18 to 1.77)	8.89	1.78	5.60 to 12.57
7	Referrals finalised on referral hub system and copy of wait list sent to clinic admin		5.00 (4.00 to 6.00)	RH: 1.01 (0.81 to 1.21)	5.06	0.72	3.74 to 6.58
	**Booking process**						
8	Register and call patient and schedule an appointment		15.83 (12.67 to 19.00)	A: 1.01 (0.81 to 1.21)	15.92	2.18	11.73 to 20.29
	**Reminder process**						
	***If appointment is booked for within 1 week***						
9	Automated SMS reminder sent day of booking		1.00 (0.80 to 1.20)	A: 1.01 (0.81 to 1.21)	1.01	0.14	0.75 to 1.29
	***If appointment is booked for after 1 week***						
10	Letter posted minimum of 1 week prior		1.00 (0.80 to 1.20)	A: 1.01 (0.81 to 1.21)	1.01	0.15	0.74 to 1.32
11	Automated SMS reminder sent 2 days prior to appointment—troubleshooting with external team		0.05 (0.04 to 0.06)	A: 1.01 (0.81 to 1.21)	0.05	0.01	0.04 to 0.06
	**Appointment attendance processes**						
	***Patient attends the appointment***						
12	Process Medicare patient		4.00 (3.00 to 5.00)	A: 1.01 (0.81 to 1.21)	4.05	0.64	2.89 to 5.38
13	Process non-Medicare patient		8.00 (6.00 to 10.00)	A: 1.01 (0.81 to 1.21)	8.09	1.29	5.66 to 10.72
	Waiting time		–	–	–	–	–
	*Musculoskeletal clinic*						
14	Initial consult (all categories)		60.00 (48.00 to 72.00)	P: 1.48 (1.18 to 1.77)	88.68	12.35	65.93 to 114.34
15	Subsequent consult	No interpreter	30.00 (24.00 to 36.00)	P: 1.48 (1.18 to 1.77)	44.61	6.11	33.18 to 57.14
16		With interpreter	60.00 (48.00 to 72.00)	P: 1.48 (1.18 to 1.77)	89.42	12.40	66.19 to 114.80
	*Women’s, men’s and pelvic health clinic*						
17	Initial consult (category 1 and 2)	No interpreter	30.00 (24.00 to 36.00)	P: 1.48 (1.18 to 1.77)	44.57	6.42	32.60 to 57.76
18		With interpreter	60.00 (48.00 to 72.00)	P: 1.48 (1.18 to 1.77)	89.21	12.37	66.41 to 114.89
19	Initial consult (category 3)		60.00 (48.00 to 72.00)	P: 1.48 (1.18 to 1.77)	89.23	12.66	66.87 to 116.50
20	Subsequent consult	Long appointment	60.00 (48.00 to 72.00)	P: 1.48 (1.18 to 1.77)	88.93	12.78	65.06 to 115.16
21		Short appointment	30.00 (24.00 to 36.00)	P: 1.48 (1.18 to 1.77)	44.32	6.09	33.19 to 56.92
	***Patient fails to attend appointment***						
22	Waiting for patient		10.00 (8.00 to 12.00)	P: 1.48 (1.18 to 1.77)	14.82	2.17	10.66 to 19.16
23	Telephone patient		11.00 (10.00 to 12.00)	P: 1.48 (1.18 to 1.77)	16.34	1.74	12.98 to 19.80
	Patient answers		–	–	–	–	–
	Patient does not answer		–	–	–	–	–
24	Convert to telephone appointment		20.00 (10.00 to 30.00)	P: 1.48 (1.18 to 1.77)	29.99	8.15	14.33 to 46.29
25	Rebook appointment and print postal letter		2.00 (1.00 to 3.00)	A: 1.01 (0.81 to 1.21)	2.02	0.57	0.94 to 3.19
26	Discharge patient		15.00 (12.00 to 18.00)	P: 1.48 (1.18 to 1.77)	22.33	3.00	16.75 to 28.50
	***Patient cancels appointment***						
27	Rebook appointment and print postal letter	No interpreter	3.00 (2.00 to 4.00)	A: 1.01 (0.81 to 1.21)	3.03	0.60	1.89 to 4.26
28		With interpreter	10.00 (8.00 to 12.00)	A: 1.01 (0.81 to 1.21)	10.10	1.45	7.19 to 12.88

Note: Displayed activity numbers correspond to numbers in figures2  3.

* Fitted to a normal distribution.

A, admin; P, physiotherapist; RH, referral hub; SMS, short message service.

Referrals can be received internally (generated within the hospital’s catchment area) or externally (generated outside the catchment area). An administrative staff member is responsible for handling internal referrals that are received via the in-house referral management system, fax, or email. This involved ensuring that each referral is directed to the appropriate physiotherapy clinic (musculoskeletal or women’s, men’s and pelvic health). External referrals are handled by the referral hub, an external department that is responsible for directing these referrals to appropriate clinics across several hospitals. External referrals are received by the physiotherapy clinics through the ambulatory referral management system, an online system designed exclusively for external referrals. A senior physiotherapist from each clinic is then responsible for triaging all referrals into categories. There are three categories based on the urgency of a patient’s referral needs. The recommended triage guidelines mandate that Category 1 patients are seen within 30 days, Category 2 within 90 days and Category 3 within 365 days.^29^ Following triage, patients are placed on the waitlist by either an administrative staff member or the referral hub. This activity is followed by the booking process, where administrative staff registers the patient, calls the patient and schedules an appointment. Appointments are not booked unless an administrative staff member has spoken to the patient.

The reminder process differs based on when an appointment is booked for a patient. Bookings within 1 week are sent short messaging service (SMS) reminders, and those booked for after 1 week are sent an SMS and a letter. For those within 1 week, the SMS reminder is organised by an administrative staff member from the physiotherapy clinic. For appointments booked after 1 week, the SMS reminder is organised by a separate team located at a different hospital (but within the same health network). The clinic’s administrative officer troubleshoots with the external team when there are issues with the SMS reminder, such as reminders not being delivered. The letter is prepared for posting to patients by an administrative staff member from the physiotherapy clinic. The referral, booking and reminder process is outlined in figure 2.

The appointment attendance process is separated into three pathways, as outlined in figure 3. Pathway A describes activities carried out for when a patient attends their scheduled appointment. Patients are billed prior to being seen by a physiotherapist. This is determined based on their Medicare status. Patients who are eligible for Medicare (funded via the government) are not billed, while billing for patients who are not eligible for Medicare is completed prior to appointment attendance. All patients are then relayed to the waiting area. Musculoskeletal patients (Clinic 1) have the same duration for an initial appointment, regardless of triage category. Women’s, men’s and pelvic health patients (Clinic 2) have varying initial appointment durations for categories 1 and 2 (30 min) and non-urgent category 3 (1 hour). Follow-up appointment duration (ie, short or long appointment) and mode (ie, telehealth, in person) varies depending on individual patient needs.

Pathway B describes staff activities for when a patient fails to attend an appointment. In the event of a patient failing to attend, the physiotherapist waits (5–10 min) before proceeding to telephone the patient. A telephone consultation can proceed if deemed appropriate, or the appointment is rescheduled (eg, unsuitable for teleconsultation; patient unavailability at the time of call). Automatic rescheduling occurs if the patient does not answer the phone call. Patients are discharged if they fail to attend two or more appointments consecutively and where it is deemed appropriate by the physiotherapist. Pathway C describes activities for when a patient cancels an appointment, which involves an administrative staff member rebooking the appointment. The time taken to rebook can depend on whether a booking for an interpreter is also required.

### Corresponding activity time

The largest portion of activity time is spent providing care to patients that attend appointments, consisting of 30–60 min during scheduled appointments (see table 1). The next highest activity time was for triaging internal referrals that are classified as a complex case. This had a higher unit of time as it was dependent on individual patient considerations such as appropriate treatment required (average of 18 min per referral). Administrative staff spent an average of 16 min booking patient appointments per referred patient and an average of 10 min rebooking cancelled appointments for those that require an interpreter.

### Individual activity costs

Individual activity costs are detailed in table 1. These costs only account for staff time relating to activities and do not include other appointment-related costs (eg, billing of appointments, equipment costs). Physiotherapists accounted for the highest portion of costs for their provision of care to patients, on average A$44 and A$89 for a short or long appointment, respectively. The next highest individual activity costs were: converting a missed appointment to a telephone appointment (on average A$30), cost of triaging (on average A$26) and cost of discharging a patient who had failed to attend more than two appointments (on average A$22). Administrative activities with the greatest activity costs were: the booking process cost (A$16 per referral) and cost to rebook cancelled appointments with an interpreter (A$10 per referral).

### Scenario costs

A scenario analysis was conducted to estimate the cost of 30 different selected bundled activities pertaining to non-attendance or cancellation of an appointment. Detailed activities and respective costs are provided in online supplemental table 2. The most expensive scenario is an internal referral with non-attendance that is converted to a telephone appointment. The mean cost was A$113 per referral for complex cases and A$90 for simple cases. Non-attendance to a rescheduled appointment for complex cases was the next most expensive scenario (mean cost A$86). The costliest process for external referrals was also non-attendance converted to a telephone appointment (mean cost A$96).

### Projected costs

The estimated non-attendance rate based on missed appointments from 2018 to 21 was 8% for Clinic 1 and 10% for Clinic 2, and the estimated cancellation rates were 4% and 8%, respectively. Given these estimates, costs were projected based on the hypothetical case assumption of all non-attendance cases or cancellation cases leading to specific outcomes, for example, assuming 100% of non-attendance to conversion to a telephone appointment. As such, the projected annual cost of non-attendance from internal referrals that are subsequently converted to a telephone appointment ranged from A$87 740–A$110 302 for outpatient Clinic 1 and A$91 340–A$114 827 for outpatient Clinic 2. The annual costs of non-attendance leading to a rescheduled appointment ranged from A$61 279–A$83 743 and A$63 793–A$87 178 for outpatient centres 1 and 2, respectively; non-attendance to discharge from A$50 593–A$93 154 and A$52 668–A$76 115; and cancellation to reschedule from A$16 508–A$31 629 and A$25 731–A$49 345. The projected costs for external referrals followed a similar pattern.

## Discussion

This study adopted the perspective of hospital clinic services and provides insight into where clinic personnel commit time in processes that are carried out in managing referrals for patients who have failed to attend a scheduled clinic appointment. Non-attendance rates at the participating clinics in this study were at the lower end of values reported in a recent scoping review (9–43%),^12^ indicating that the impact of non-attendance at clinics participating in the present study may be a conservative estimate in comparison to similar clinics with higher non-attendance rates. Nonetheless, substantial healthcare resource waste was evident in both participating hospital outpatient clinics despite comparatively low rates of non-attendance. These findings have highlighted the extent to which non-attendance at scheduled clinic appointments may not only impact patients’ welfare through lost opportunity to receive treatment, but also substantial opportunity cost burden from wasted resources that could have been otherwise allocated to other referred patients.

Key opportunities for reducing costs were identified in the present study. This included potentially exploring alternatives to current systems used within the clinic, such as the separate systems used for similar processes (eg, paper-based internal referrals vs electronic external referrals) and the duplicative reminder system processes. The clinic also lacked an integrated booking system. These factors influencing cost estimates are unlikely to be isolated to the hospital clinics participating in the present study, and as such, there is likely to be substantial opportunity for similar health services in Australia or internationally to improve processes and implement solutions to reduce resource waste. Prior research has indicated the potential for interventions, including automated reminder systems, non-attendance prediction models coupled with a targeted intervention, telehealth and advanced booking systems, to reduce the impact of non-attendance at clinic appointments.^1230^,^32^ Clinics participating in the present study were already employing common non-attendance reducing strategies, including reminder systems and the ability to convert a missed inperson attendance to a teleconsultation where appropriate. However, it is noteworthy that this latter strategy still carried substantial time burden, and in the context of physiotherapy intervention delivery, not all interventions are easily amenable to a teleconsultation.

Findings from the present study must be interpreted with caveats. This study was conducted at two co-located physiotherapy clinics serving patients from the same geographical region, and while findings may potentially generalise to other clinics with similar characteristics (ie, other publicly-funded physiotherapy clinics or those with similar non-attendance rates), it may not generalise to dissimilar hospitals or populations. Nonetheless, given that non-attendance is potentially faced by healthcare providers across varying systems and settings, the findings from this study may be useful for other clinics considering investigating the impact of non-attendance within their clinic. Workflow process maps were suitable for addressing the study aims, but may need to be adapted to reflect relevant activities constituting key clinic processes for other hospital clinics. The scope of this study was limited to understanding only the personnel labour costs (productivity costs) as the resource use associated with each activity of a process. The inclusion of other resources such as consumables may be relevant in other clinical contexts. Further, workflow process maps and time estimates were generated using facilitated stakeholder discussions followed by a verification activity. An uncertainty analysis was conducted as part of this study to account for potential variations and uncertainty in reported estimates. However, contextual observations or the use of a mobile application designed for activity time measurement, in addition to facilitated stakeholder discussions, may provide further validation of time estimates but were beyond the scope of feasibility for the present study.^33^ Additional research in this field is required to explore cost-effectiveness, preferences for and acceptability of implemented solutions for reducing non-attendance to help inform resource allocation efforts towards enhancing high-value service delivery. Scope also remains for further exploration of staff and patient experiences and perceptions related to non-attendance at hospital clinic appointments that may assist with providing insight on barriers and enablers to attendance that may contribute to actionable solutions for supporting patient-centred care with further reduction in non-attendance. It is also noteworthy that this study was not designed with a control or comparison study arm to examine comparative effectiveness, and we cannot make definitive statements regarding the labour costs as entirely undesirable or inefficient. Nonetheless, it must be noted that highlighting these costs provides useful information for health system administrators and decision makers seeking to improve the efficiency of hospital outpatient clinic systems.

## Conclusion

Non-attendance is a complex behaviour, and outpatient services like those examined in the present study adapt a range of labour-intensive strategies to mitigate this issue. However, the efficacy of implemented processes remains unclear, despite the accrual of significant labour costs. Evidence-based interventional processes may optimise non-attendance mitigation strategies that release substantial labour resources to engage in productive activities. This study also highlights the importance of further research in the field, including exploration of patients’ and staff perceptions of barriers and enablers of missed appointments, which is likely to provide valuable insights regarding the appropriateness or otherwise of potential solutions for reducing the impact of non-attendance in the context of hospital outpatient clinic appointments.

## Supplementary material

10.1136/bmjopen-2023-083420 online supplemental appendix 1

10.1136/bmjopen-2023-083420 online supplemental table 1

## Data Availability

The datasets generated and analysed during the current study are available upon reasonable request. Other data relevant to the study has been included in the article or uploaded as supplemental material.
