# Supplementary material for: Cost of physiotherapy non-attendance at a metropolitan hospital in Australia: A time-driven activity-based costing study
Source: BMJ Open. 2025 May 24;15(5):e083420. doi: 10.1136/bmjopen-2023-083420 (PMC12104927; doi:10.1136/bmjopen-2023-083420)
Supplement: online supplemental appendix 1 [file bmjopen-15-5-s001.pdf]

Supplementary Material

Supplementary File 2

Interview Guide – Process Map Creation and Obtaining Time Estimates

DESCRIPTION OF STEPS FOR PROCESS MAP CREATION AND OBTAINING OF TIME ESTIMATES

Process map creation (TDABC step 1) and obtaining time estimates (TDABC step 2) were conducted concurrently. This involved two semi-structured facilitated discussions conducted via videoconference with key stakeholders (n=3) to create process maps and obtain time estimates. This was followed by a validation activity conducted via email correspondence with the broader staff team from both clinics, which included 11 physiotherapists and 4 administrative staff. All participants were involved in at least one of the following clinic procedures: (1) operationalising the clinic referral process, (2) appointment scheduling, and (3) provision of care to patients.

HOW TO USE THIS GUIDE

The questions presented in this guide provide a general overview of possible questions that can be used to facilitate the process of creating workflow process maps and obtaining time estimates for each activity constituting main clinic processes. Within the context of this study, the aim is to understand how staff spend their time performing various tasks associated with managing non-attendance at their clinic.

The interviewer should follow the questions and prompts from the section(s) of the guide. Within each section, remember:

1. It is not necessary for interviewers to ask questions in the proposed order, or ask all questions exactly as stated, or repeat a question if it has been answered in an earlier section.
2. Encourage the participant to think about all possible variations and exceptions in activities and associated timings.
3. Interviewers will ensure discussion progresses in a timely, yet informative manner.

INTERVIEW QUESTIONS

Facilitated Discussions:

1. Could you describe the main activities or processes you perform in your role?
    a. Could you break down each process into specific steps or tasks?
    b. Could you briefly describe what you do during this step?

2. How long does it typically take you to complete each task or step?
    a. How many hours/minutes in a day do you typically spend performing this task?
    b. What is the shortest amount of time it would take to complete an activity (in hours/minutes)?
    c. What is the longest amount of time it would take to complete an activity (in hours/minutes)?

3. Are there any variations in the time required for these tasks depending on different factors (e.g., appointment type (in-person vs telehealth))?
    a. If so, what are the variations in the time required to complete these tasks?

4. Do you find that there are instances where there are any idle times or waiting periods between tasks?

5. How often (frequent) do you perform each activity in a given time period (e.g., daily, weekly, monthly)?

6. Are there any unusual or infrequent tasks that might not be captured in a typical workweek?

Validation Activity:

Within the context of this study, participants were presented with the process maps (displayed as visual figures) and time estimates (displayed in a table format) generated from the facilitated discussions. All participants were asked to verify if any component was either missing or inaccurate using the following potential prompts:

- Please refer to the presented figures and table and verify if the activities and corresponding time estimates are accurate.
    - What are your thoughts on the sequence of activities presented?
    - Are any activities or time estimates incorrect?
    - Are any activities or time estimates missing?

REFERENCES

- Kaplan RS, Witkowski M, Abbott M, Guzman AB, Higgins LD, Meara JG, Padden E, Shah AS, Waters P, Weidemeier M, Wertheimer S. Using time-driven activity-based costing to identify value improvement opportunities in healthcare. Journal of Healthcare Management. 2014 Nov 1;59(6):399-412.
- McBain RK, Jerome G, Warsh J, Browning M, Mistry B, Faure PA, Pierre C, Fang AP, Mugunga JC, Rhatigan J, Leandre F. Rethinking the cost of healthcare in low-resource settings: the value of time-driven activity-based costing. BMJ global health. 2016 Nov 1;1(3):e000134.

End of guide
