# Supplementary material for: Cost of physiotherapy non-attendance at a metropolitan hospital in Australia: A time-driven activity-based costing study
Source: BMJ Open. 2025 May 24;15(5):e083420. doi: 10.1136/bmjopen-2023-083420 (PMC12104927; doi:10.1136/bmjopen-2023-083420)
Supplement: online supplemental table 1 [file bmjopen-15-5-s002.pdf]

Supplementary Material

Supplementary File 1

Supplemental Table 1. Estimation of practical capacity cost rates for labour cost categories

Estimation of practical capacity cost rates (CCR) for labour cost category				
Cost category	Midpoint Annual Salary ($)	Salary + 25% Oncosts ($)	Practical capacity (hours)	Capacity cost rate ($/hour) *Mean (Range)*
Administration officer	$67,569	$84,461.25	1392	$60.68 ($48.54–$72.81)
Physiotherapist	$98,641	$123,301.25	1392	$88.58 ($70.86–$106.29)
NOTE: Practical capacity cost rate is estimated to be 80%.				

Supplemental Table 2. Costs and actions included for scenario analysis

Scenario	Referral processing		Case complexity		Referral activities			Reminder process		Non-attendance activities					Rebook cancelled appt		Scenario cost (AUD) Mean (95% CI)*
	Process internal referral	Process external referral	Triage: simple case	Triage: complex case	Triage: external referral	Wait listed patients	Booking appointment	Within one week	After one week	Waiting for patient	Telephone patient	Convert appointment	Rebook appointment	Discharge patient	No interpreter	With interpreter	
**Activity Number**	**1**	**5**	**2**	**3**	**6**	**4 / 7**	**8**	**9**	**10,11**	**22**	**23**	**24**	**25**	**26**	**27**	**28**	
**Internal**																	
Non-attendance convert appointment to telehealth	✓	-	✓	-	-	✓	✓	✓	-	✓	✓	✓	-	-	-	-	89.99 (70.41–111.82)
	✓	-	✓	-	-	✓	✓	-	✓	✓	✓	✓	-	-	-	-	90.04 (70.44–111.92)
	✓	-	-	✓	-	✓	✓	✓	-	✓	✓	✓	-	-	-	-	113.08 (85.18–143.80)
	✓	-	-	✓	-	✓	✓	-	✓	✓	✓	✓	-	-	-	-	113.13 (85.34–143.92)
Non-attendance to reschedule appointment	✓	-	✓	-	-	✓	✓	✓	-	✓	✓	-	✓	-	-	-	62.79 (52.93–73.31)
	✓	-	✓	-	-	✓	✓	-	✓	✓	✓	-	✓	-	-	-	62.85 (53.09–73.36)
	✓	-	-	✓	-	✓	✓	✓	-	✓	✓	-	✓	-	-	-	85.89 (63.81–108.10)
	✓	-	-	✓	-	✓	✓	-	✓	✓	✓	-	✓	-	-	-	85.94 (63.90–108.13)
Non-attendance to discharge	✓	-	✓	-	-	✓	✓	✓	-	-	-	-	-	✓	-	-	51.89 (43.43–61.42)
	✓	-	✓	-	-	✓	✓	-	✓	-	-	-	-	✓	-	-	51.94 (43.59–61.61)
	✓	-	-	✓	-	✓	✓	✓	-	-	-	-	-	✓	-	-	74.98 (54.21–96.67)
	✓	-	-	✓	-	✓	✓	-	✓	-	-	-	-	✓	-	-	75.03 (54.31–96.58)
Cancel to reschedule	✓	-	✓	-	-	✓	✓	✓	-	-	-	-	-	-	✓	-	32.82 (26.02–40.22)
	✓	-	✓	-	-	✓	✓	-	✓	-	-	-	-	-	✓	-	32.87 (26.15–40.42)
	✓	-	✓	-	-	✓	✓	✓	-	-	-	-	-	-	-	✓	39.79 (31.64–48.10)
	✓	-	✓	-	-	✓	✓	-	✓	-	-	-	-	-	-	✓	39.85 (31.71–48.25)
	✓	-	-	✓	-	✓	✓	✓	-	-	-	-	-	-	✓	-	55.91 (36.57–75.29)
	✓	-	-	✓	-	✓	✓	-	✓	-	-	-	-	-	✓	-	55.96 (36.68–75.38)
	✓	-	-	✓	-	✓	✓	✓	-	-	-	-	-	-	-	✓	62.88 (42.93–82.67)
	✓	-	-	✓	-	✓	✓	-	✓	-	-	-	-	-	-	✓	62.94 (42.83–82.75)
**External**																	
Non-attendance to convert appointment	-	✓	-	-	✓	✓	✓	✓	-	✓	✓	✓	-	-	-	-	96.28 (76.20–117.57)
	-	✓	-	-	✓	✓	✓	-	✓	✓	✓	✓	-	-	-	-	96.33 (76.25–117.41)
Non-attendance to reschedule appointment	-	✓	-	-	✓	✓	✓	✓	-	✓	✓	-	✓	-	-	-	68.89 (57.68–80.83)
	-	✓	-	-	✓	✓	✓	-	✓	✓	✓	-	✓	-	-	-	68.94 (57.67–81.02)
Non-attendance to discharge	-	✓	-	-	✓	✓	✓	✓	-	-	-	-	-	✓	-	-	58.08 (48.23–68.41)
	-	✓	-	-	✓	✓	✓	-	✓	-	-	-	-	✓	-	-	58.13 (48.36–68.47)
Cancel to reschedule	-	✓	-	-	✓	✓	✓	✓	-	-	-	-	-	-	✓	-	38.82 (31.63–47.02)
	-	✓	-	-	✓	✓	✓	-	✓	-	-	-	-	-	✓	-	38.88 (31.66–47.08)
	-	✓	-	-	✓	✓	✓	✓	-	-	-	-	-	-	-	✓	45.87 (37.48–55.06)

	-	✓	-	-	✓	✓	✓	-	✓	-	-	-	-	-	-	✓	45.92 (37.52–55.07)

*Fitted to a normal distribution
