# Supplementary material for: Cost of physiotherapy non-attendance at a metropolitan hospital in Australia: A time-driven activity-based costing study
Source: BMJ Open. 2025 May 24;15(5):e083420. doi: 10.1136/bmjopen-2023-083420 (PMC12104927; doi:10.1136/bmjopen-2023-083420)
Supplement: Supplementary file 3 [file bmjopen-2023-083420.reviewer_comments.pdf]

PEER REVIEW HISTORY

BMJ Open publishes all reviews undertaken for accepted manuscripts. Reviewers are asked to complete a checklist review form and are provided with free text boxes to elaborate on their assessment. These free text comments are reproduced below.

ARTICLE DETAILS

Title (Provisional)

Cost of physiotherapy non-attendance at a metropolitan hospital in Australia: a time-driven activity-based costing study

Authors

Mohammed Selim, Shayma; Naicker, Sundresan; Kularatna, Sanjeewa; Carter, Hannah; Borg, Samantha; Armstrong, Codie; Walkenhorst, Melanie; Kunst, Brittney; McPhail, Steven M

VERSION 1 - REVIEW

**Reviewer**	**1**
**Name**	**Jiang, Zekun**
**Affiliation**	**Sichuan University**
**Date**	**13-Feb-2024**
**COI**	**None.**

In my view, this is an interesting research work, presenting a refreshing topic that, at least for me, is the first of its kind I have encountered. For my review, the work obtained data from relevant hospitals for analysis, examined the expenses, attendance, and management of related departments, and drew some interesting conclusions, providing guiding suggestions. The figures and tables are good, and the work has also obtained ethical approval, making the entire research relatively comprehensive.

Of course, these are the most basic requirements for a publishable work, though I might not be very familiar with the research background. My question is, do the results and conclusions of our study have referential value for hospitals in Australia or other countries? Are there any commonalities and guidelines at the hospital management level?

From another perspective, can we draw general conclusions by only studying data from a specific hospital? This involves whether our title itself needs to be modified. Perhaps appropriately revising and narrowing the research topic could better ensure publication. Additionally, no significant issues were found elsewhere; please consider making modifications based on the feedback from other reviewers.

**Reviewer**	**2**
**Name**	**Kim, NamKwen**
**Affiliation**	**Pusan National University Korean Medicine Hospital**
**Date**	**19-Feb-2024**
**COI**	**I have no conflict of interest.**

This manuscript is the research to estimate the costs associated with non-attendance of out-patient clinic using TDABC method. This study might be valuable to estimate the healthcare burden of non-attendance and discussed the impact from the societal perspective. I recommend the author to revisit and add descriptions about the vague points in some parts which are as follows.

Revision request.

1. Please add the statistical analysis part describing normality test of continuous variables and analysis program used in this study or other related descriptions .

2. In p.8(Step 6 Uncertainty analysis) To my knowledge, the author might use Bootstrapping sampling(simulation) to determine 95% confidence interval, and also use Monte Carlo simulation for conducting probabilistic sensitivity analysis.

3. Please describe the variables and their distributions (beta or gamma etc.) and parameters (mean or alpha etc.) you conducted Monte Carlo simulation in the Table.

VERSION 1 - AUTHOR RESPONSE

Review 1 Comments	Response
In my view, this is an interesting research work, presenting a refreshing topic that, at least for me, is the first of its kind I have encountered. For my review, the work obtained data from relevant hospitals for analysis, examined the expenses, attendance, and management of related departments, and drew some interesting conclusions, providing guiding suggestions. The figures and tables are good, and the work has also obtained ethical approval, making the entire research relatively comprehensive.	

Of course, these are the most basic requirements for a publishable work, though I might not be very familiar with the research background.<br><br>My question is, do the results and conclusions of our study have referential value for hospitals in Australia or other countries? Are there any commonalities and guidelines at the hospital management level?	Non-attendance has remained an ongoing concern for many healthcare providers globally. However, while there have been estimates of wasted healthcare resources attributable to non-attendance reported in international literature (e.g., UK and USA), the costs to the Australian health system has received little exploration in existing literature. As such, this study sought to contribute findings that have referential values for health services in Australia, but which may also be relevant to other health services in industrialised countries. The hospital where the study was conducted is quite typical in characteristics for metropolitan hospitals in Australia, and other similar healthcare systems in high income countries.<br><br>In this manuscript, we have now ensured we provide a more complete overview of the study context throughout the manuscript, including the following sections of text in the introduction:<br><br>*"…Although there is no current robust national estimate of wasted healthcare resources attributable to non-attendance in Australia, there have been compelling estimates arising from healthcare systems internationally. For example, in the United Kingdom, the projected non-attendance costs across the entirety of the healthcare system is estimated to be £912 million per annum.[3] Estimated non-attendance costs across the United States healthcare system equate to US$150 billion per annum.[4]"* (Lines 5-10)<br><br>*"A recent scoping review identified highly variable rates of non-attendance at health appointments in the international published literature, ranging from 9% to 43%.[12] Two studies identified in that review reported non-attendance rates in Australia. Both studies were specific to dental clinics, with one identifying a non-attendance rate of 9.3%[13], while the other identified non-attendance at 5% among adults and 11% among children.[14] Yet, even with these non-attendance rates, the costs to the health system have received little exploration in existing literature.[2] Furthermore, it has been recognised that to foster improved value in healthcare, when conceptualised as health outcomes achieved per dollar spent, it is imperative to first understand where resource waste is occurring.[15]"* (Lines 24-31)<br><br>We also sought to provide context for the interpretation of the results and conclusions (presented in the discussion section of the manuscript), accounting for the potential that other health service providers may have similar characteristics (e.g., similar non-attendance rates) or employ similar processes (e.g., similar reminder system):
From another perspective, can we draw general conclusions by only studying data from a specific hospital? This involves whether our title itself needs to be modified. Perhaps appropriately revising and narrowing the research topic could better ensure publication. Additionally, no significant issues were found elsewhere; please consider making modifications based on the feedback from other reviewers.	

	"Non-attendance rates at the participating clinics in this study were at the lower end of values reported in a recent scoping review (9% to 43%)[12] *indicating the impact of non-attendance at clinics participating in the present study may be a conservative estimate in comparison to similar clinics with higher non-attendance rates."* (Lines 283-286)<br><br>*"Key opportunities for reducing costs were identified in the present study. This included potentially exploring alternatives to current systems used within the clinic such as the separate systems used for similar processes (e.g., paper-based internal referrals versus electronic external referrals), and the duplicative reminder system processes. The clinic also lacked an integrated booking system. These factors influencing cost estimates are unlikely to be isolated to the hospital clinics participating in the present study, and as such, there is likely to be substantial opportunity for similar health services in Australia or internationally to improve processes and implement solutions to reduce resource waste."* (Lines 293-299)<br><br>Accounting for the recommendations, we have amended the limitations section of the manuscript to provide improved clarity surrounding the extent to which general conclusions can be drawn from the findings of this study:<br><br>*"Findings from the present study must be interpreted with caveats. This study was conducted at two co-located physiotherapy clinics serving patients from the same geographical region and while findings may potentially generalise to other clinics with similar characteristics (i.e., other publicly- funded physiotherapy clinics or those with similar non-attendance rates), it may not generalise to dissimilar hospitals or populations. Nonetheless, given that non-attendance is potentially faced by healthcare providers across varying systems and settings, the findings from this study may be useful for other clinics considering investigating the impact of non-attendance within their clinic."* (Lines 309- 315)<br><br>We have also amended the title of the study to better reflect the setting of the study (having been conducted in Australia):<br><br>*"Cost of physiotherapy non-attendance at a metropolitan hospital in Australia: A time-driven activity-based costing study"*

**Review 2 Comments**	**Response**
This manuscript is the research to esbmate the costs associated with non-acendance of out-pabent clinic using TDABC method. This study might be valuable to esbmate the healthcare burden of non-acendance and discussed the impact from the societal perspecbve. I recommend the author to revisit and add descripbons about the vague points in some parts which are as follows.	

1. Please add the stabsbcal analysis part describing normality test of conbnuous variables and analysis program used in this study or other related descripbons.	We think there may have been a misunderstanding here, which we hope our further descripbon has clarified. In our study, we did not do a normality test of conbnuous variables as our model inputs were based on a parametrised esbmate and range that represented a consensus among all study informants from parbcipabng clinics. Data collecbon was conducted at two physiotherapy outpabent clinics co-located within a single hospital, and the following process was used to obtain model inputs used to esbmate total costs:<br>• Process map creation (TDABC step 1) and obtaining time estimates (TDABC step 2) were conducted concurrently. This involved two semi-structured facilitated discussions conducted via videoconference with key stakeholders (n=3) to create process maps and obtain consensus on time estimates. This was followed by a validation activity conducted via email correspondence with the broader staff team from both clinics, which included 11 physiotherapists and 4 administrative staff. All activities and corresponding time estimates were reported by staff.<br>• Costing resources (TDABC step 3): Resources were costed based on Australian currency (AUD) for July 2023 and were relevant to the health service where the study was being conducted. Personnel labour rates were calculated using the mid-point of the 2023 award rates for the state of Queensland for physiotherapists and administrative staff. The labour cost estimates included salary and an additional 25% on-costs to account for leave and other entitlements including superannuation funded by the health service.<br><br>To account for potenbal variabons and uncertainty in reported esbmates, an uncertainty analysis was conducted as part of this study. We have amended the descripbon of the process for conducbng the uncertainty analysis in the manuscript to provide clarity:<br><br>*“Step 6: Uncertainty analysis*<br>*Monte Carlo simula[ons were performed to account for uncertainty associated with the TDABC model inputs ([me es[mates and labour cost) used in es[ma[ng the total costs generated in Step 5. To reflect uncertainty surrounding the values used for es[mates of staff [me for conduc[ng different ac[vi[es and the varying labour costs for different personnel, a probability distribu[on was assigned to each parameter. In this study, a normal distribu[on was assumed for each parameter. ShinyPrior, a web-based applica[on for es[ma[ng probability distribu[ons from summary sta[s[cs was used to convert es[mates for each model input elicited during the facilitated discussion into a distribu[on (mean and standard devia[on) that could be used in the simula[ons.[28] The Monte Carlo simula[ons was conducted using 1000 itera[ons, in which values were randomly sampled from the probability distribu[on constructed for each model input to generate simulated observa[ons. These simulated observa[ons were then used to es[mate the mean and 95% confidence intervals for the total cost of each ac[vity.”* (Lines 157-169)

	Addibonally, we have added the following statement to the manuscript indicabng the analysis program used:<br><br>*"All analyses including Monte Carlo simula[ons were performed in Microsob Excel."* (Lines 177-178)
2. In p.8(Step 6 Uncertainty analysis) To my knowledge, the author might use Bootstrapping sampling(simulabon) to determine 95% confidence interval, and also use Monte Carlo simulabon for conducbng probabilisbc sensibvity analysis.	The revisions made in response to the above comment have likely also offered clarificabon regarding this comment also. For the 'process map creabon and obtaining bme esbmates for each acbvity presented in the maps' (TDABC step 1 and 2) component of data collecbon – consensus was reach amongst key stakeholders (n=3) for a single esbmate represenbng the most likely minimum (lower range) and maximum bme (upper range) to complete each acbvity. The process maps and corresponding bmes esbmates were then presented as a figure and table to the broader clinic staff team (all staff including key stakeholders (n=15)) to validate esbmates (i.e., staff verified and confirmed that the presented single esbmate per acbvity accurately represents bme taken within their clinic to complete tasks).<br><br>To provide clarity surrounding the process for obtaining a consensus on bme esbmates – we have amended the btle for step 1 and 2, and the descripbon around obtaining bme esbmates:<br><br>*"Steps 1 and 2: Process map crea[on and obtaining consensus [me es[mates"* (Line 100)<br><br>*"The second facilitated discussion focused on verifying the key workflow processes and corresponding ac[vi[es outlined in the process map. A [me es[mates for each ac[vity was simultaneously obtained during this verifica[on process. Clinic staff were asked to es[mate how long (in minutes) each ac[vity took (e.g., [me taken by an administra[ve staff member to book an appointment) and reach a group consensus on each es[mate. To account for variability in [me taken for specific ac[vi[es, par[cipants also discussed and agreed on a minimum [me and maximum [me to complete each ac[vity. Subsequently, process maps were finalised with [me es[mates and presented for valida[on to the broader clinic staff team. To conduct the valida[on ac[vity, we used a think-aloud method to ensure that the data used in TDABC calcula[ons were appropriate and represented up-to-date organisa[onal prac[ces.[26] The [me es[mates elicited for each ac[vity and associated minimum/maximum values were presented in a table format, to aid in their comprehensibility when think-aloud[26] took place, while the process maps were represented as visual figures for a more accessible interpreta[on. All par[cipants were asked to verify if any component was either missing or inaccurate. Following this collabora[ve review, a study researcher (SMS) made any necessary adjustments to both the process maps and their corresponding [me es[mates, verbally relaying this informa[on back to par[cipants for a final round of verifica[on. The same study researcher (SMS) then finalised both process maps and the corresponding [me es[mates."* (Lines 116-132)

	In addibon, we have amended the descripbon for the process followed for conducbng the uncertainty analysis in the manuscript:<br><br>*"Step 6: Uncertainty analysis*<br>*Monte Carlo simula[ons were performed to account for uncertainty associated with the TDABC model inputs ([me es[mates and labour cost) used in es[ma[ng the total costs generated in Step 5. To reflect uncertainty surrounding the values used for es[mates of staff [me for conduc[ng different ac[vi[es and the varying labour costs for different personnel, a probability distribu[on was assigned to each parameter. In this study, a normal distribu[on was assumed for each parameter. ShinyPrior, a web-based applica[on for es[ma[ng probability distribu[ons from summary sta[s[cs was used to convert es[mates for each model input elicited during the facilitated discussion into a distribu[on (mean and standard devia[on) that could be used in the simula[ons.[28] The Monte Carlo simula[ons was conducted using 1000 itera[ons, in which values were randomly sampled from the probability distribu[on constructed for each model input to generate simulated observa[ons. These simulated observa[ons were then used to es[mate the mean and 95% confidence intervals for the total cost of each ac[vity."* (Lines 157-169)
3. Please describe the variables and their distribubons (beta or gamma etc.) and parameters (mean or alpha etc.) you conducted Monte Carlo simulabon in the Table.	In addibon to the amendment to the descripbon of the process for conducbng the uncertainty analysis (described above), and accounbng for the recommendabon, we have amended the tables. The table footnote now displays the fit distribubon (normal distribubon), and the total cost secbon now includes a new header indicabng Monte Carlo Simulabons were conducted to generate the cost. The total cost secbon now also includes the standard deviabon results (in addibon to the mean and 95% CI).
